# HER2 expression and relevant clinicopathological features in gastric and gastroesophageal junction adenocarcinoma in a Chinese population

**DOI:** 10.1186/1746-1596-8-76

**Published:** 2013-05-09

**Authors:** Ling Shan, Jianming Ying, Ning Lu

**Affiliations:** 1 Department of Pathology, Cancer Hospital, Peking Union Medical College & Chinese Academy of Medical Sciences, Beijing, China

**Keywords:** HER2, Gastric cancer, Gastroesophageal junction adenocarcinoma, Chineses, Clinicopathological features

## Abstract

**Background:**

With varied immunohistochemistry scoring criteria and patient cohorts, HER2-positivity rates in gastric cancer (GC) and gastroesophageal junction (GEJ) adenocarcinoma have been reported with a wide range. Recently standardized scoring criteria for GC and GEJ cancer has been established and recommended. In this study, the frequency of HER2 expression and the relationship between HER2 expression and clinicopathological features were examined in a large cohort of Chinese GC and GEJ cancer patients.

**Methods:**

A total of 1463 patients, including 929 primary GCs and 534 primary GEJ adenocarcinomas, was retrospectively analyzed for HER2 overexpression by immunohistochemistry (IHC). Fluorescence *in situ* hybridization (FISH) analysis was used in 308 GCs and GEJ adenocarcinoma cases to assess *HER2* gene amplification.

**Results:**

HER2 overexpression (3+) was detected in 9.8% of carcinomas and more frequently observed in GEJ cancer cases, in the intestinal type, and in the well or moderately differentiated type (*P*=0.003, 0.000, and 0.000, respectively). HER2 equivocal (2+) was detected in 14.4% of cases. As for the 308 cases analyzed by FISH, 39 (of 40, 97.5%) IHC 3+ cases, 11 (of 38, 28.9%) IHC 2+ cases, and 3 (of 230, 1.3%) IHC 1+/0 cases showed *HER2* gene amplification. A high concordance rate (98.5%) between IHC and FISH was demonstrated.

**Conclusions:**

Approximately 10% of Chinese patients with primary GC and GEJ adenocarcinoma were HER2-positive on IHC. HER2 overexpression was associated with GEJ site, intestinal cancer subtype, and well or moderately differentiated carcinomas.

**Virtual slides:**

The virtual slide(s) for this article can be found here: http://www.diagnosticpathology.diagnomx.eu/vs/1935951199941072.

## Background

Gastric cancer (GC) is the fourth most commonly diagnosed cancer and the second most common cause of cancer-related deaths worldwide [1]. It has been suggested that the incidence rate of gastroesophageal junction (GEJ) adenocarcinoma was increased in recent years [2,3]. Despite some advances in the prevention and treatment of gastric cancer, 5-year survival remains around 20% in most parts of the world. HER2 is now well recognized as a key factor in the development of certain solid human tumors, most notably in breast cancer. *HER2* gene amplification and protein overexpression, which occur in 20% to 25% of breast cancer patients, have been recognized as prognostic and predictive markers for treatment [4]. Multiple detection methods have been established to examine *HER2* gene status and protein expression [5-8]. Trastuzumab, a recombinant monoclonal antibody targeting HER2 protein, is now being applied not only in metastatic breast cancer cases but also to localized cases as adjuvant therapy [9,10]. A recent phase III randomized study (ToGA) revealed that combination treatment with trastuzumab and chemotherapy significantly improved survival in patients with advanced GC or GEJ cancer with HER2 overexpression [11]. Thus, trastuzumab was recently approved for the treatment of metastatic adenocarcinomas of the stomach and GEJ in many countries [12-17].

Although many studies have previously evaluated HER2 status in GC, the patient cohorts and scoring criteria have varied, resulting in discrepancies in HER2-positivity rates varying from about 4% to 53%, with a median rate of 18% [18]. The ToGA study developed a new set of IHC scoring criteria based on the study by Hofmann et al. [19] and found HER2-positive (defined as IHC 3+ or IHC 2+/FISH+) tumors in 16% of metastatic GC cases. The efficacy of trastuzumab for treating metastatic GC with HER2 overexpression demonstrated in the ToGA study is also promising for resectable HER2-positive gastric cancer. However, few studies have been conducted to examine the frequency of HER2-positive tumors determined by the new criteria in resectable gastric cancer [20,21], especially in a large Chinese cohort. In this study, IHC analysis according to standardized scoring criteria was used to assess the incidence of HER2-positivity in primary resected GC and GEJ cancer samples in a C9pt?>The relationship between HER2 overexpression and gene amplification was also examined in GC and GEJ adenocarcinoma.

## Methods

### Samples

A total of 1,463 patients with primary GC or GEJ adenocarcinoma, who received curative surgery (no history of neoadjuvant chemotherapy) in the Cancer Institute & Hospital, Chinese Academy of Medical Sciences (CICAMS), Beijing, China, between August 2009 and February 2012, was included in this retrospective study. All tumor samples were fixed in 10% neutral buffered formalin for 24–48 h and embedded in paraffin, and routinely diagnosed in the Department of Pathology, CICAMS, Beijing. The study protocol was approved by the Institutional Review Board (IRB). The patients’ medical records were reviewed to obtain patients’ clinicopathological parameters, including age at diagnosis, sex, tumor location, histological classification, and pathological TNM stage. Histological classification was determined according to the World Health Organization (WHO) classification and Lauren’s classification.

### Immunohistochemistry

Automated IHC was performed on 4-μm-thick sections using an automated slide stainer, the Ventana Benchmark XT (Ventana Medical Systems, Tucson, AZ, USA), according to the manufacturer’s instructions, for the Ventana CONFIRM™ HER2/neu (4B5) Rabbit Monoclonal Primary Antibody. HER2 IHC was scored using the scoring scheme proposed by Hofmann et al. [19] in the ToGA cohort of gastric cancer (ToGA score) and Ruschoff et al. [22] as follows: 0, no staining or membranous reactivity in <10% of tumor cells; 1+, faint/barely perceptible membranous reactivity in ≥10% of tumor cells (cells are reactive only in part of their membrane); 2+, weak to moderate complete, basolateral, or lateral membranous reactivity in ≥10% of tumor cells; and 3+, complete, basolateral, or lateral membranous reactivity of strong intensity in ≥10% of tumor cells.

Samples scoring IHC 0 or IHC 1+ were considered negative for HER2 overexpression, whereas samples scoring IHC 3+ were considered positive for HER2 overexpression. Samples scoring IHC 2+ were considered equivocal for HER2 overexpression.

### Fluorescence *in situ* hybridization

Fluorescence *in situ* hybridization (FISH) analysis was carried out with the PathVysion HER2 DNA probe kit and procedures (Vysis/Abbott, Abbott Park, IL, USA). The kit contains two fluorochrome-labeled DNA probes, LSI HER2 (labeled with SpectrumOrange) and CEP17 (chromosome 17 enumeration probe, labeled with SpectrumGreen). Pretreatment was carried out with the Paraffin Pretreatment Kit (VysisAbbott). The HER2 signals and CEP17 signals of 20 nuclei of invasive tumor cells in two different areas were counted using a Zeiss AxioImager M2 epifluorescence microscope (Carl Zeiss, Oberkochen, Germany) equipped with an ×100 oil immersion objective and 4’,6’-diamidino-2-phenylindole (DAPI)/Spectrum Green/Orange single and triple bandpass filters. The HER2/chromosome 17 ratios were interpreted as follows: a HER2/CEP17 ratio higher than 2.2 was defined as amplification of the *HER2* gene, while a ratio <1.8 was defined as no amplification of the *HER2* gene. When the ratio was between 1.8 and 2.2, signals in another 20 nuclei were counted, and the HER2/CEP17 ratio in a total of 40 nuclei was determined. When the ratio was ≥2.0, it was defined as amplification of the *HER2* gene; otherwise it was defined as no amplification of the *HER2* gene.

### Statistical analysis

Statistical analysis was performed using the chi-square test to analyze associations between HER2 status and clinicopathological parameters. A *P* value less than 0.05 was considered significant. Data were analyzed using the SPSS statistical software program for Microsoft Windows (SPSS Inc., Chicago, IL, USA).

## Results

### HER2 overexpression and clinicopathological characteristics

A total of 1,463 patients was evaluated by IHC analysis. The patients’ clinicopathological characteristics are listed in Table 1. Their median age was 58 years (range, 20 to 82 years), with a male predominance (75.5%). Of all patients, there were 650 cases (44.4%) of intestinal type by Lauren’s classification, 564 cases (38.6%) of diffused type, and 249 cases (17%) of mixed type. In terms of tumor location, approximately two-thirds of tumors were primary gastric cancers (63.5%), and one-third was primary GEJ adenocarcinomas (36.5%). The tumors were poorly differentiated in 73.1%, moderately differentiated in 25.2%, and well-differentiated in 1.7%. Overall, most patients were at pathologic stage T3 (73.4%), with 14.1%, 10%, and 2.5% at stage T1, T2, and T4, respectively. Regarding the pathologic N stage, there was an approximately equal distribution of patients at N0, N1, N2, or N3 stages (28.1%, 16.1%, 20.9%, 34.9%, respectively). The majority of patients (97.7%) had no distant metastases.

**Table 1:** The correlation between HER2 expression status and clinicopathological parameters

	**Overall**	**HER2**	**HER2-**	**HER2-**	
		**Overexpression**	**Equivocal**	**Negative**	***P*value**
**Pathologic feature**	**n=1463**	**n=143 (%)**	**n=211 (%)**	**n=1109 (%)**	
**Age at diagnosis**					0.102
≥60 years	683	80 (11.7)	108 (15.8)	495 (72.5)	
<60 years	780	63 (8.1)	103 (13.2)	614 (78.7)	
**Sex**					0.344
Male	1104	109 (9.9)	171 (15.5)	824 (74.6)	
Female	359	34 (9.5)	40 (11.1)	285 (79.4)	
**Tumor location**					0.003
GC	929	65 (7.0)	121 (13.0)	743 (80.0)	
GEJ	534	78 (14.6)	90 (16.9)	366 (68.5)	
**Tumor subtype**					
**(Lauren classification)**					0.000
Intestinal	650	109 (16.8)	111 (17.1)	430 (66.2)	
Diffuse	564	13 (2.3)	63 (11.2)	488 (86.5)	
Mixed	249	21 (8.4)	37 (14.9)	191 (76.7)	
**Tumor differentiation**					0.000
Well	25	4 (16)	9 (36)	12 (48)	
Moderately	369	74 (20.1)	70 (19.0)	225 (61.0)	
Poorly	1069	65 (6.1)	132 (12.3)	872 (81.6)	
**pT status**					0.053
pT1	206	16 (7.8)	33 (16.0)	157 (76.2)	
pT2	147	23 (15.6)	28 (19.0)	96 (65.3)	
pT3	1074	102 (9.5)	145 (13.5)	827 (77.0)	
pT4	36	2 (5.6)	5 (13.9)	29 (80.6)	
**pN status**					0.074
pN0	411	39 (9.5)	66 (16.1)	306 (74.5)	
pN1	235	27 (11.5)	42 (17.9)	166 (70.6)	
pN2	306	20 (6.5)	42 (13.7)	244 (79.7)	
pN3	511	57 (11.2)	61 (11.9)	393 (76.9)	
**pM status**					0.571
pM0	1429	140 (9.8)	119 (8.3)	1080 (75.6)	
pM1	34	3 (8.8)	2 (5.9)	29 (85.3)	
**pTNM stages**					0.327
IA	162	14 (8.6)	29 (17.9)	119 (73.5)	
IB	93	8 (8.6)	17 (18.3)	68 (73.1)	
IIA	201	25 (12.4)	33 (16.4)	143 (71.1)	
IIB	230	25 (10.9)	34 (14.8)	171 (74.3)	
IIIA	264	15 (5.7)	37 (14.8)	212 (80.3)	
IIIB	455	51 (11.2)	56 (12.3)	348 (76.5)	
IIIC	24	2 (8.3)	3 (12.5)	19 (79.2)	
IV	34	3 (8.8)	2 (5.9)	29 (85.3)	

**Figure 1:**
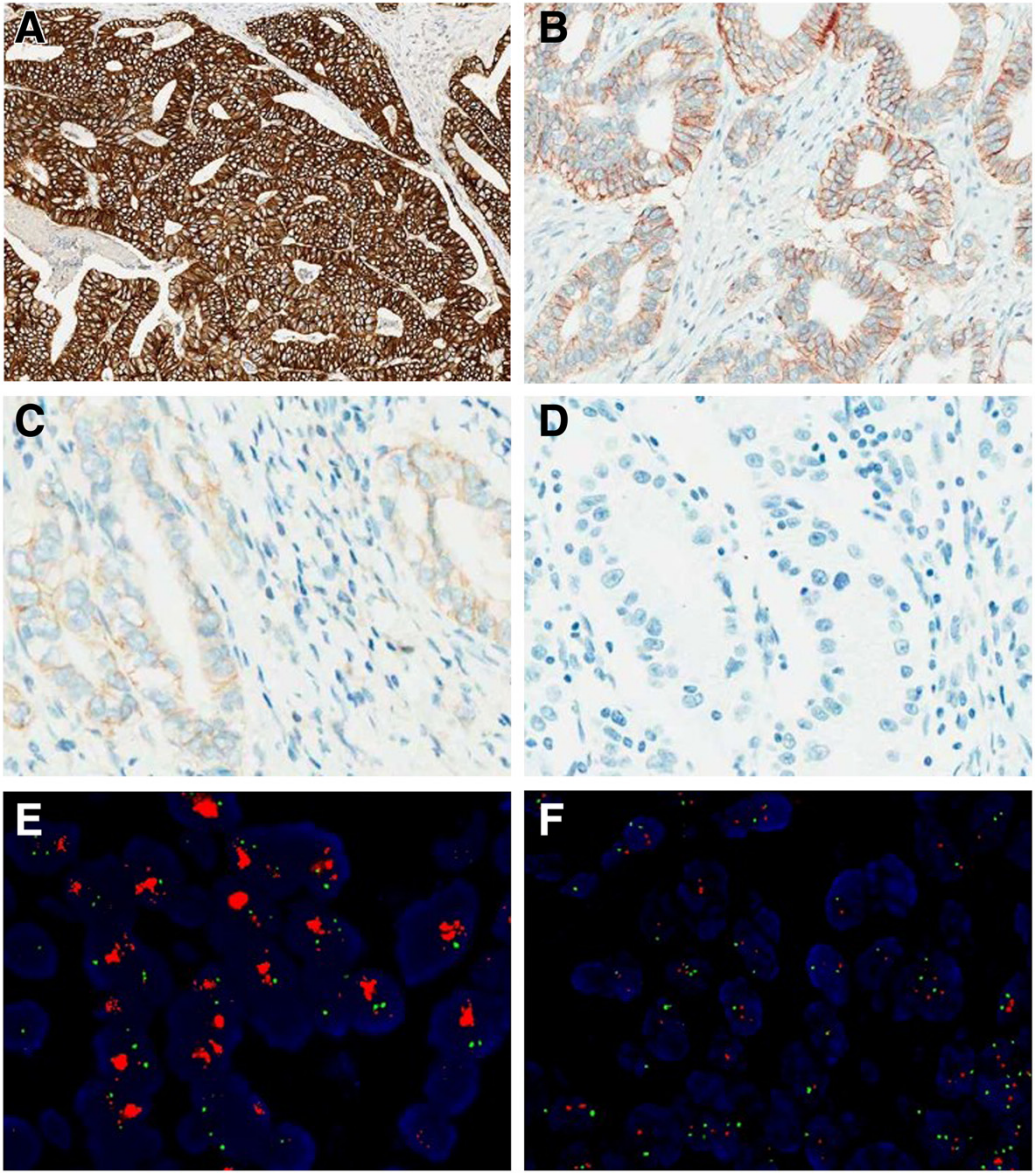
**Representative cases of IHC staining and FISH analysis in GC and GEJ adenocarcinoma. A**. HER2 IHC staining shows complete, basolateral, or lateral membranous reactivity of strong intensity (3+) in tumor cells (original magnification, ×40). **B**. HER2 IHC staining shows weak to moderate complete, basolateral, or lateral membranous reactivity (2+) in tumor cells (original magnification, ×100). **C**. faint/barely perceptible membranous reactivity (1+) in tumor cells (original magnification, ×200). **D**. no staining (0) in tumor cells (original magnification, ×200). **E**. a GC case with HER2 amplification using FISH analysis. Green signals refer to the reference probe of Chr. 17 centromere, while red signals are the target probe for *HER2*. **F**. A GC case without HER2 amplification using FISH analysis.

Of the 1463 patients, 143 (9.8%) patients were scored as HER2 overexpression (3+), while 211 (14.4%) and 1109 (1463, 75.8%) were equivocal and negative, respectively. Table 1 summarizes HER2 expression status by subgroup. HER2 was overexpressed (score 3+) in 7.0% (65/929) of GC cases and 14.6% (78/534) of GEJ adenocarcinoma cases (Figure 1A). Equivocal expression (2+) of HER2 protein was observed in 13.0% (121/929) of GC cases and 16.9% (90/534) of GEJ adenocarcinoma cases (Figure 1B). HER2 was negative (1+/0) in 80.0% (743/929) of GC cases and 68.5% (366/534) of GEJ adenocarcinoma samples (Figure 1C and D). A significant difference in HER2 overexpression was found between GCs and GEJ adenocarcinomas (7.0% vs. 14.6%, *P*<0.05). HER2 overexpression was associated with the intestinal-type carcinomas by the Lauren classification (*P*<0.001): 16.8% in intestinal vs. 2.3/8.4% in diffuse-/mixed-type cancers. HER2 overexpression was also correlated with well or moderately differentiated carcinomas according to the WHO classification. No correlation was observed between HER2 overexpressed and not overexpressed cases in terms of age, sex, pT, N, M factors, or pathologic TNM stage.

### Concordance of HER2 IHC and FISH

FISH analysis was performed in 308 randomly selected GC and GEJ adenocarcinoma cases, including 40 cases with HER2 overexpression (IHC 3+), 38 cases with equivocal (IHC 2+), and 230 cases with negative (IHC 1+/0) HER2 expression. *HER2* amplification was observed in 97.5% (39/40) of HER2 IHC 3+ cases, 28.9% (11/38) of HER2 IHC 2+ cases, and 1.3% (3/230) of HER2-negative (1+/0) cases, showing a concordance rate of 98.5% between IHC and FISH (Table 2) (Figure 1E and F).

**Table 2:** Correlation between HER2 overexpression and gene amplification in GC and GEJ adenocarcinoma

**FISH**		**IHC**		**Total**
	**3+**	**2+**	**1+/0**	
Amplification	39 (97.5%)	11 (28.9%)	3 (1.3%)	53
No amplification	1(2.5%)	27 (71.1%)	227 (98.7%)	255
Total	40	38	230	308

## Discussion

In this study, 143 of 1463 (9.8%) of GC and GEJ adenocarcinoma cases were HER2-positive (3+) by IHC in one of the largest Chinese studies to date. In the ToGA trial, the percentage of HER2-positive (IHC 3+ or IHC 2+/FISH positive) GC or GEJ cancer patients was 22.1% overall and around 10.4% of IHC 3+ in resected samples [23], similar to the present result. Recent studies also reported a range of 8.5% to 10.3% for HER2 overexpression in GC [20,21,24]. Recently, three studies in Chinese GC cases applied the same FDA-approved reagents and scoring criteria and reported HER2 IHC 3+ rates of 9.0% (77/860) [25], 6.9% (10/145) [26], and 5.8% (4/69) [27], respectively, all slightly lower than the present result. This variation may be partly explained by different sample sets. In addition, HER2-positivity varied by tumor site, with higher rates of HER2-positivity in GEJ adenocarcinoma than in stomach cancer in this study (14.6% vs. 7.0% respectively; *P*<0.01), which is consistent with the results of other studies [11,20]. In the ToGA trial, the countries with the highest ratio of GEJ:stomach cancer were found to have above-average HER2-positivity rates, regardless of sample size [23]. Another explanation is the different ratio of intestinal:diffuse/mixed cancer among the studies. A positive association between HER2-positivity and intestinal-type cancers was identified in this and other studies [28-33]. In the ToGA trial, for example, countries with higher ratios of intestinal:diffuse/mixed cancer had increased HER2-positivity rates [23].

No correlation was found between HER2 overexpression and pT, N, or M factors, or TNM stages, in the present study. Previous studies including all pathological stages have also reported no correlation between pathological stage and overexpression [20,21,24]. A correlation of HER2-positivity with well or moderately differentiated carcinomas was found in the present study (*P*<0.01). However, other studies demonstrated both an association and no association between HER2 overexpression and tumor differentiation [25,32,34,35]. These conflicting data may be due to different sample sizes and the low prevalence of HER2 in GC and GEJ adenocarcinoma. In addition, varying methods of evaluation and scoring schemes with different cut-points were used before the establishment of a standard guideline for assessing HER2 expression. Perhaps with the current consistent guidelines for HER2 assessment in this disease, future studies will provide more clarity regarding this issue.

The present study revealed a high concordance rate, 98.5%, between IHC and FISH, which is similar to another study of surgical resections from 145 Chinese GC cases, with a concordance rate of 94.5% [26]. In contrast, in the ToGA study, the concordance rate of IHC and FISH was 87.2%, as 7.5% of IHC 1+/0 samples and 54.6% of IHC 2+ cases were found to be FISH-positive [23]. One of the possible explanations for this discrepancy might be different specimen types and reagents. In the ToGA study, both biopsies and surgically-resected specimens were included; the HER2-positivity rate was higher in biopsies than in surgically-resected specimens (23.1% vs. 19.9%; P=0.03), and biopsy samples were also more likely to be HER2-amplified than surgical samples when analyzed by FISH (P=0.01) than by IHC [23]. The majority of current studies using a standard guideline for HER2 expression assessment used biopsies and tissue microarrays (TMAs), which were tested analogous to biopsies, to interrogate HER status in GC. The concordance rate of IHC and FISH in these studies varied from 88.6% to 97.7% [20,32,36]. A specific study evaluating HER2 protein expression on whole-tissue sections and TMAs was conducted, indicating a discrepancy in HER2 expression on whole-tissue sections from TMAs. For instance, the proportion of IHC 2+ was 4.7% in whole-tissue sections, whereas it was 8.6% in TMAs, which might be partially explained by heterogeneous HER2 expression [33]. Another possibility, which was revealed by a study aiming to validate the guideline for HER2-expression assessment in GC, was different interpretations of staining of TMAs among observers, although a uniform assessment guideline was followed [22]. Therefore, reliable separation of IHC 1+/0 and IHC 2+ may be difficult in biopsy samples, and FISH analysis should be used for definitive classification.

In conclusion, a thorough analysis of 1463 Chinese GC/GEJ cancer cases using FDA-approved reagents showed HER2 overexpression in 9.8% of carcinomas, with a strong preference for GEJ location, intestinal cancer subtype, and well or moderately differentiated carcinomas. Approximately 29% of IHC 2+ cases showed *HER2* gene amplification, and there was a high concordance rate (98.5%) between IHC and FISH in GC and GEJ adenocarcinoma.

## Competing interests

None of the authors has an affiliation or conflict of interest.

## Authors’ contributions

Study concept and design: JY, NL. Analysis and interpretation of data: LS, JY. Drafting of the manuscript: LS, JY. All authors read and approved the final manuscript.
